# Potential antidepressant effects of a dietary supplement from the chlorella and lion's mane mushroom complex in aged SAMP8 mice

**DOI:** 10.3389/fnut.2022.977287

**Published:** 2022-09-02

**Authors:** Ming-Yu Chou, Jou-Hsuan Ho, Mao-Jung Huang, Ying-Ju Chen, Mei-Due Yang, Liang-Hung Lin, Ching-Hsin Chi, Chin-Hsi Yeh, Tsui-Ying Tsao, Jian-Kai Tzeng, Rachel Jui-cheng Hsu, Ping-Hsiu Huang, Wen-Chien Lu, Po-Hsien Li, Ming-Fu Wang

**Affiliations:** ^1^International Aging Industry Research & Development Center (AIC), Providence University, Taichung, Taiwan; ^2^Department of Food Science, Tunghai University, Taichung, Taiwan; ^3^School of General Education, Hsiuping University of Science and Technology, Taichung, Taiwan; ^4^Ph.D. Program in Health and Social Welfare for Indigenous Peoples, Providence University, Taichung, Taiwan; ^5^Department of Surgery, Department of Clinical Nutrition, China Medical University Hospital, Taichung, Taiwan; ^6^Division of Allergy, Immunology & Rheumatology, Taichung Tzu Chi Hospital, Buddhist Tzu Chi Medical Foundation, Taichung, Taiwan; ^7^Taiwan Chlorella Manufacturing Co., Ltd., Taipei, Taiwan; ^8^China Grain Products Research and Development Institute, New Taipei, Taiwan; ^9^College of Food, Jiangsu Food and Pharmaceutical Science College, Huai'an City, China; ^10^Department of Food and Beverage Management, Chung-Jen Junior College of Nursing, Health Sciences and Management, Chia-Yi City, Taiwan; ^11^Department of Food and Nutrition, Providence University, Taichung, Taiwan

**Keywords:** antidepressant, chlorella, lion's mane mushrooms, senescence accelerated mouse prone-8 (SAMP8), dietary supplement

## Abstract

Since the 1990s, the prevalence of mental illnesses, such as depression, has been increasing annually and has become a major burden on society. Due to the many side effects of antidepressant drugs, the development of a complementary therapy from natural materials is an urgent need. Therefore, this study used a complex extract of chlorella and lion's mane mushroom and evaluated its antidepressant effects. Six-month-old male senescence-accelerated mice prone-8 (SAMP8) were divided into positive control; negative control; and low, medium, and high-dose groups. All groups were treated with corticosterone (CORT) at 40 mg/Kg/day for 21- days to induce depression in the animals, and the effects of different test substances on animal behavior was observed. The positive control group was intraperitoneally injected with a tricyclic antidepressant (Fluoxetine, as tricyclic antidepressant), the control group was given ddH_2_O, and the test substance groups were administered test samples once daily for 21 days. The open field test (OFT) and forced swimming test (FST) were applied for behavior analyses of depression animal models. The OFT results showed that the mice in the positive control and the medium-, and high-dose groups demonstrated a significantly prolonged duration in the central area and a significantly increased travel distance. In the FST, the positive control and the medium, and high-dose groups displayed significantly reduced immobility times relative to the control group. The blood analysis results showed significant decreases in triglyceride and blood urea nitrogen levels relative to the positive control and the medium- and high-dose groups. Notably, in the positive control and the medium- and high-dose groups, brain-derived neurotrophic factor (BDNF) increase by more than in the control group. In summary, medium and high dose of extract of chlorella and lion's mane mushroom could improve depression behavior in animals and have the potential to be antidepressant health care products.

## Introduction

Nowadays, depression is a prevalent psychiatric disorder that seriously impairs the quality of human life. Roughly 12% of people experience depression at least once in their lifetime (1). Approximately 32% of cancer patients suffer from anxiety, depression, or adjustment disorders (2). Depression is a global emotional disorder that affects an estimated 350 million people worldwide (3). It is characterized by a psychiatric disorder that can be caused by neuroendocrine dysfunction with disabling symptoms, usually depressed mood, despair, and low curiosity, with high morbidity, disability, and mortality, seriously endangering human life and health. In preclinical and clinical studies, it has been found that exogenous stress may cause a negative feedback imbalance in the hypothalamic-pituitary-adrenal (HPA) axis, promoting the release of glucocorticoids in the body (3). The glucocorticoid, also known as corticosterone (CORT), has been described as a “stress hormone” recognized as a mediator between chronic stress and depression. Currently, the widely used CORT-induced depression-like behavior was based on the neuroendocrine model of stress theory (4). It was reported that repeated injections of CORT to mice resulted in a time-dependent rise in immobility in forced swimming and tail suspension tests. Simultaneously, this injection regularization produced time-related effects on tyrosine hydroxylase levels in the hippocampus of mice; these results are consistent with the relevance of a stress-induced depression model and indicate that repetitive corticosterone injection regularization provides a valuable and reliable mouse model (5). Even though various antidepressants have been used to treat depression, a significant proportion of patients do not respond to them, and some experience side effects.

Recently, the marine microalga chlorella (*Chlorella vulgaris*) has been available as a dietary supplement and marketed worldwide (6). Its applications include the food, pharmaceutical, and agriculture industries (7). Chlorella has been proven to have various pharmacological effects on animals and humans and has a well-established production chain and commercial product line (8). It was documented by the FDA as safe for human consumption (9). Moreover, according to a previous published study, male and female mice dosed orally with acute and repeated doses of chlorella demonstrated no toxicity or adverse effects at levels estimated at 1,000 mg/kg^−1^ body weight per day and did not die during the period of investigation (10). Available commercially, chlorella products contain nutrients essential for humans (notably, vitamin D2 and B12), high amounts of high-quality protein, dietary fiber, and polyunsaturated fatty acids (including α-linoleic and linoleic acids) (1, 11, 12). In addition, chlorella possesses valuable antioxidants such as chlorophyll, carotenoids, astaxanthin, total polyphenols, lutein, and phycobiliproteins (13–15). However, a study has shown that chlorella extract (1,800 mg/day) was well-tolerated in a human clinical trial in major depression, with no serious adverse events reported; moreover, the participants exhibited improved physical and cognitive symptoms of depression (16). It has been proposed that antioxidant nutrients and compounds are responsible for the therapeutic effect of chlorella on depression (1).

A popular saprotrophic fungus in Asia (primarily in China, Taiwan, and Japan), the lion's mane mushroom [*Hericium erinaceus* (Bull.) Pers.] is a source of health-promoting properties and nutrients, including dietary fiber, minerals, vitamins, and bioactive compounds that have beneficial effects on human health, such as β-glucan, a fungal polysaccharide with health-promoting anti-tumor and immune-stimulating properties (17–19). Specifically, the *in vivo* benefits of mushroom antioxidants include reduced lipid peroxidation (20), reduced postprandial triglyceride response (21), improved activity of antioxidant enzymes (superoxide dismutase and catalase, etc.), increased plasma antioxidant capacity (T-AOC), protection against oxidative stress, removal of non-radioactive electrophiles (e.g., hydrogen peroxide), and breakdown of superoxide anions (22). Ultimately, it would appear that antioxidants play a role in limiting or reducing cellular and neurological damage in neurodegenerative diseases such as Alzheimer's and Parkinson's disease (23). Other physiological activities contain potential therapeutic applications in oxidative stress-related diseases such as atherosclerosis, cancer, cardiovascular disease, inflammation, and diabetes (22).

Specifically, a role of oxidative stress in the pathophysiology of depression has been identified. Thus, alternative antidepressant drugs with adequate efficacy and safety are needed. This study aimed to observe the effect of different doses of a complex extract of chlorella and lion's mane mushroom on depression behavior in SAMP8 mice.

## Materials and methods

### Materials

Concentrated extracted of chlorella (*Chlorella Vulgaris*) was purchased from Taiwan Chlorella Manufacturing Co. (Taipei City, Taiwan), and the total polyphenols (expressed as gallic acid equivalents), as a quality control indicator component, were 23.31–34.93 (mg GAE/g extract). Lion's mane mushroom (*Hericium erinaceus* (Bull.) Pers.) was purchased from a local market. It was washed with distilled water, wiped dry, and then frozen at −20°C for 24 h, followed by freeze-drying. The powder was dry milled in a homogenizer (through an 80-mesh sieve). Next, 3.5 L of 95% alcohol and 210 g of mushroom powder were placed in a 5 L flask. The flask was shaken every 4 h for 24 h. The obtained solution was labeled as “extract A.” The alcohol was removed by rotary vacuum evaporation. The filtered mushroom powder mentioned above was then added to 3.5 L of ddH_2_O for 24 h and shaken at least four times. The solution resulting from this step was labeled “extract B.” The precipitate was filtered out and added to a pot containing 10.5 L of water, which was then heated to 80°C. It was kept at this temperature and stirred every 6 h for ~36 h until the solution volume was reduced to 3.5 L. The solution was labeled as “extract C.” While the decoction was cooling, the mushroom powder was filtered out and discarded/composted. The three extract solutions (designated extracts “A,” “B,” and “C”) were combined. The final extract was obtained by rotary vacuum evaporation and concentrated to 100 mL. Acceptable quality was indicated by polysaccharide> 12% and β-glucan> 7%. The tricyclic antidepressant Fluoxetine, trade name Prozac® (each capsule contains 20 mg, Eli Lilly and Company, Indianapolis, Indiana, USA), was applied and purchased from a local hospital according to the animal experiment's apply code in accordance with the regulation.

### Experimental animal

A total of 40 male, 6-month-old SAMP8 mice were purchased from the National Laboratory Animal Center (Taipei, Taiwan) and randomly distributed into positive control, negative control, and treatment groups (low, medium, and high doses of extract), for a total of five groups of eight mice each (*n* = 8). Additionally, a group of 6-month-old BALB/C mice served as the blank group (n= 8). The mice were housed under specific pathogen-free conditions (25°C ± 2°C, humidity of 65% ± 5%, 12 h light/dark cycle, and lights on at 7 p.m.) with diet (AIN-93M standard purified feed) and water supplied *ad libitum* during the experiment. Feed and drinking water were freshly prepared and replaced every other day in the morning. All animal procedures were conducted in accordance with the standards set forth in the guidelines for the Care and Use of Experimental Animals by the Committee for the Purpose of Control and Supervision of Experiments on Animals and the National Institutes of Health. The protocol was approved by the Committee on Animal Research, Providence University, under code 20170512-A01.

### Design of animal experiments

According to a related study, the spontaneous behavior is more active when mice is in the dark period; therefore, all tests were performed during the dark period (24). All groups were subjected to an open field test (OFT) and forced swimming test (FST) before the experiments. On the first day of the experiment, all groups of mice were injected subcutaneously with corticosterone (CORT) (40 mg/ kg/day) for 21 days to induce depression. In this case, CORT was dissolved in saline (NaCl 0.9 %) containing 0.1% dimethyl sulfoxide (DMSO) and 0.1% Tween-80, followed by subcutaneous injection once daily at 0.05 mL/10 g body weight (BW) per treatment (25).

The following were given 30 min after CORT injection.

i. ddH_2_O was given to both the BALB/c and the negative control group.
ii. Fluoxetine (10 mg/kg, *ig*) was given to the positive control group.
iii. In the low-dose group, extract was given at 0.25 mL/25 g BW/day via tube feeding. The complex extract ratio was 0.1 mL chlorella+ 6 mg lion's mane mushroom.
iv. The medium-dose group was given 0.5 mL/25 g BW/day via tube feeding. The complex extract ratio was 0.2 mL chlorella + 12 mg lion's mane mushroom.
v. The high-dose group was given 2.5 mL/25 g BW/day via tube feeding. The complex extract ratio was 0.4 mL chlorella+ 24 mg lion's mane mushroom.

**Figure 1:**
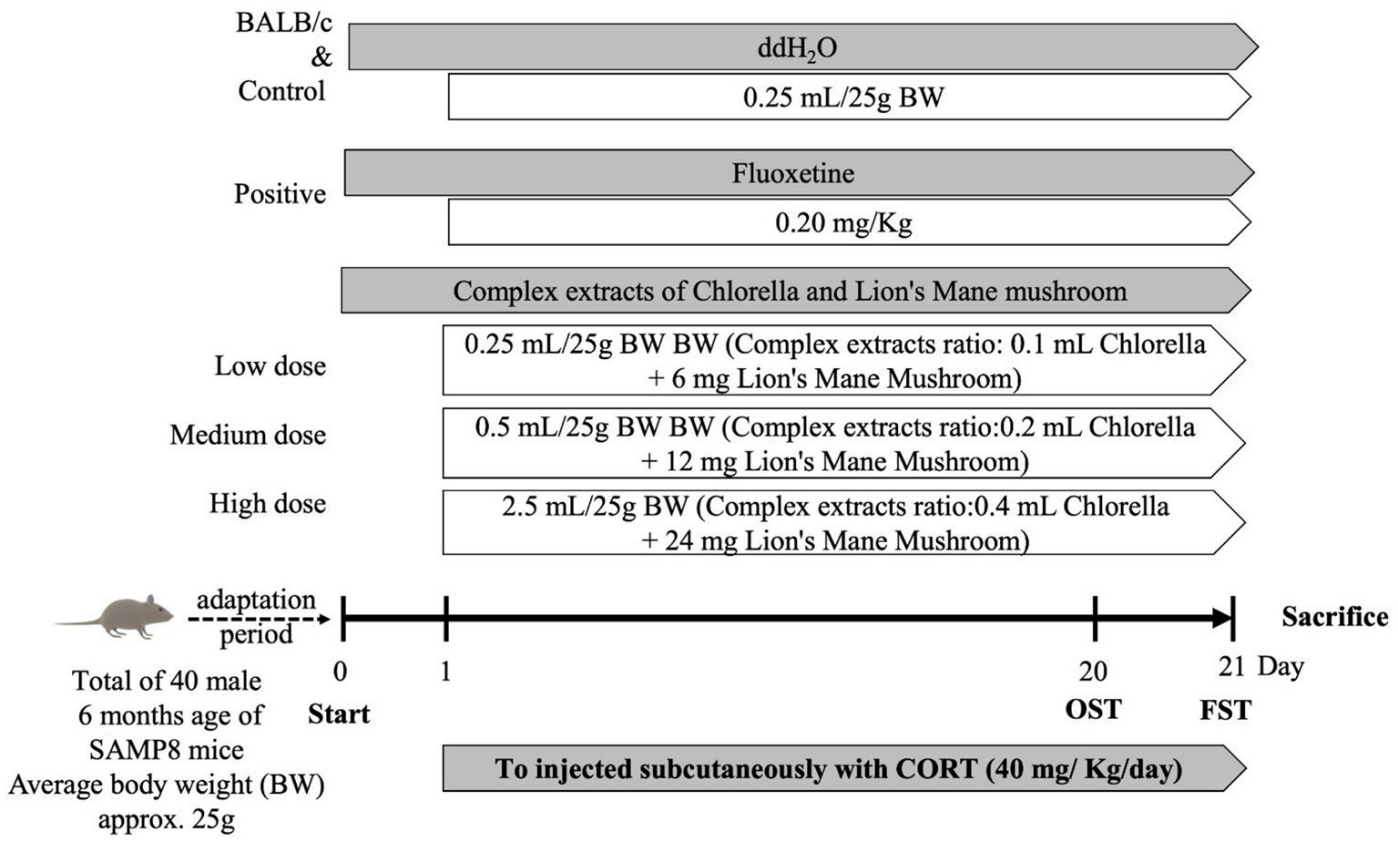
Schedule of animal experiments. Random subgroup adaptation for 7 days followed by treatment of mice with combined extracts, Fluoxetine, or water for 21 consecutive days, respectively. All mice were exposed to the CORT procedure for 21 days. Behavioral tests were performed on all mice.

The study duration was 21 days (Figure 1), and BW, food intake, and water intake were recorded. The degree of depression in mice was evaluated by OFT and FST on days 20 and 21, respectively. After behavioral testing was completed, animals were anesthetized with isoflurane before dissection, while their blood was used for biochemical analysis. Whole-brain tissue was rinsed with N_2_ and stored at −80°C for further analysis of factors associated with the central nervous system.

### Open field test (OFT)

The evaluation methodology was based on previous studies, with slight modifications (26–28). Rodents display characteristic of thigmotaxis to a new environment. Therefore, the central zone represents a threatening situation, while the peripheral area is relatively safe. Experiments were conducted in a location shielded from incidental noise. The space is a circular arena with a diameter of 97 and 42 cm high walls. Lighting with 850 nm far-red LEDs was used and any anxiety induced by the bright illumination was minimized. The room temperature was maintained at 25°C± 2°C during data acquisition. Each mouse was recorded for 10 min and then returned to the group housing. Mice were observed by video recording (FDR-AX700, Sony, Tokyo, Japan), and the time spent in the center, frequency of entering the center, and time spent standing were recorded for 10 min. An increase in the frequency and time spent staying in the center of the space indicated a decrease in anxiety. Video analysis was performed with TruScan software (Version 2.2; Coulbourn Instruments, Allentown, PA, USA). After each test, the space was cleaned with a 70–75% alcohol solution to avoid the effect of the odor of the previous animal on the next one.

### Forced swimming test (FST)

The FST capitalizes on the fact that animals display escape behavior when exposed to adverse conditions, and when efforts to struggle fail, they present a state of abandonment. The FST, as a measure of depression, was conducted as in published studies, with modifications (29–31). The FST test was performed after 21 days of continuous gavage. The specific experimental procedure was as follows: 30 min after the last gavage, each mouse was placed individually and gently from a height of 5 cm into an open glass cylinder (20 cm diameter and 30 cm height) filled with warm water (25°C ± 2°C); under these conditions, the mice initially swam in the water to maintain their stability. Each mouse was judged to be immobile when it stopped struggling and remained floating motionless in the water, making only the movements necessary to keep its head above the water. The process was videotaped for 6 min to observe the behavior of each mouse. The time spent on activity (mobility) and inactivity (immobility) during the last 4 min of the 6 min were recorded to assess the degree of depression. The longer the duration of immobility, the higher the degree of desperation. In this case, the increase in immobility time corresponded to depression, and a decrease in immobility time was evaluated as the effectiveness of antidepressant treatment. When each mouse was finished, the test apparatus was cleaned with a 70–75% ethanol solution. To minimize experimental error, when each mice completed the test, the water was changed and the test apparatus cleaned with 70–75% ethanol.

### Biochemical analysis of blood

Immediately after animal sacrifice, the blood was centrifuged at 4°C and 12,000 g for 10 min in Microfuge 22R (Beckman Coulter Inc., Brea, Calif., USA). Next, the plasma biochemical parameters, including glucose, total protein, albumin, triglycerides, total cholesterol, GOT (Glutamate Oxaloacetate Transaminase), GPT (Glutamate Pyruvate Transaminase), BUN (Blood urea nitrogen), and creatinine were analyzed in the blood using the Synchroh LX-20 system (Beckman Coulter Inc., Brea, Calif., USA), according to the manufacturer's instructions.

### Analyses of factors associated with the central nervous system in the brain

Whole-brain tissues were homogenized with Tris-buffer (pH 7.4) and centrifuged at 1,200 g for 10 min at 4°C by removing unbroken tissues and debris. The tissue homogenate was obtained by centrifugation again at 13,000 g for 10 min at 4°C to prepare the cytoplasmic fraction.

#### Serotonin (5-HT)

Serotonin concentrations were quantified using the Serotonin Research ELISA^TM^ (Labor Diagnostika Nord GmbH & Co.KG, Nordhorn, Germany) and an ultra-sensitive enzyme immunoassay. Determination was performed according to the protocol in the reagent kit. Briefly, 100 μL of tissue homogenate and standard were added to the acylation plate, then 25 μL of acylation buffer was added at room temperature to react for 30 min. From the above-acetylated samples and standards, 100 μL were transferred to a 96-well dish, to which 25 μL of serotonin antiserum was added and then placed in a refrigerator at 4°C for 15–20 h. Then, after three washes with a Wellwash™ (Thermo Fisher Scientific Co., Waltham, MA, USA), 100 μL of IgG-peroxidase conjugate (anti-rabbit) was added and allowed to react at room temperature for 30 min, and the above washing procedure was repeated. The reaction was performed by adding 100 μL of TMB at room temperature for 20 to 30 min without light and then terminated by adding 100 μL of 25 M sulphuric acid. The absorbance values of the samples and standards were determined at 450 nm (Spectrophotometer, U-2000, Hitachi High-Tech Science, Tokyo, Japan), and the serotonin concentrations were calculated by comparison with standard curves.

#### Dopamine (DA)

Quantification of dopamine was performed using the Dopamine Research ELISA^TM^ (Labor Diagnostika Nord) and an ultra-sensitive enzyme immunoassay as follows: 10 μL of tissue homogenate and standards were added to the extraction plate and diluted with 90 μL of deionized water. Then, 25 μL of TE buffer was added and incubated for 1 h at room temperature. After two washes, 150 μL of acylation buffer and 25 μL of acylation reagent were added and allowed to react at room temperature for 20 min. The reaction was repeated by washing twice and adding 100 μL of 0.025 M HCl to react for 10 min at room temperature. Then, 90 μL was transferred from the extraction plate to a 96-well microtiter plate, 25 μL of enzyme solution was added, and the plate was incubated at 37°C for 2 h. Then, 100 μL of the reaction solution was transferred to the dopamine ELISA plate and incubated in the refrigerator at 4°C for 15–20 h. Wellwash™ was used to wash the plate three times, and 100 μL of IgG-peroxidase conjugate (anti-rabbit) was added and allowed to react for 30 min at room temperature. The washing procedure was repeated. Under sheltered conditions, 100 μL of TMB was added to the reaction at room temperature for 20–30 min, then 100 μL of 0.25 M H_2_SO_4_ was added to stop the reaction. The absorbance values were measured at 450 nm, and the concentration of dopamine was calculated by comparison with a standard curve.

#### Brain-derived neurotrophic factor (BDNF)

The *ChemiKine* BDNF Sandwich ELISA Kit (Merck KGaA, Darmstadt, Germany) was used to determine BDNF by using an enzyme immunoassay. Tissue homogenates and standards (100 μL) were added to a 96-well plate and incubated overnight in a 4°C refrigerator. Wellwash™ was used to wash the plate four times. Then, 100 μL of anti-BDNF (from mouse) was added and allowed to react at room temperature for 2.5 h. Following triple washing, 100 μL of the streptavidin-enzyme conjugate was added, and the reaction was carried out at room temperature for 1 h. The plate was washed three more times and 100 μL of TMB added and allowed to react for 15 min at room temperature without light. Lastly, the reaction was terminated by adding 100 μL of HCl, and the absorbance values were measured at 450 nm, while the content of BDNF in the samples was calculated based on comparison with the standard curve.

### Statistical analysis

The data obtained from this study were analyzed using IBM SPSS Statistics version 22 (IBM Corp., Armonk, N.Y., USA). The results were expressed as mean ± SEM. The data were analyzed by one-way analysis of variance (ANOVA) to test for differences between groups, and Duncan's multiple allometric tests were used to assess differences between groups for significance. When *p* < 0.05, a significant difference was indicated.

## Results and discussion

### Changes in diet, water intake, and BW

Stress hormones such as CORT play a vital role in the development of depression (32, 33), and this relationship has been used to develop animal models of the condition (25, 34, 35). The CORT-induced depression model has a shorter test schedule, with a higher repeatability and success rate, for rapid assessment of antidepressant efficacy, enabling an expedient evaluation, and has been utilized in many studies (5, 33, 35, 36). In depressive mood disorders, continuous changes in the HPA axis are observed, usually associated with stress (32). Therefore, this study induced dysregulation of the HPA axis in male SAMP8 mice by repeated injections of CORT (40 mg/kg/day), which led to depression and anxiety. Improvements were evaluated and observed when mice were fed different doses of combined extracts of chlorella and lion's mane mushroom for 21 consecutive days. A comparison of the changes in BW, food intake, and water intake in all groups is shown (Table 1). To minimize experimental error, the mice were randomized into groups with no significant difference in initial BW. The results showed no significant differences in BW, food intake, and water intake between any of the groups from the beginning to the end of the experiment. However, each group showed a slight decrease in BW during the experimental period. In addition, the food and water intake of the control group showed an increasing trend, in comparison with the other groups. It was observed that mice injected with 40 mg/kg of CORT for 21 consecutive days showed a reduction in BW compared to the control group (37). Moreover, when CORT was added directly to the rats' drinking water, the CORT group consumed more food and water than the control group at weeks 2 and 3, while the bodyweight of the CORT group was significantly lower than that of the control group at weeks 1, 2, and 3 (38). Additionally, a previous study found that glucocorticoids act on the hypothalamus to promote appetite and increase food intake (39). The administration of glucocorticoids (such as CORT) increases catabolic processes, including lipid and muscle protein catabolism (36). Therefore, when food and water intake increase, weight loss still occurs. The above reports showed that the BW of the animals decreased with CORT administration, and the food and water intake increased. In this study, decreases in BW were observed in the positive control group, negative control group, and experimental group, but only the negative control group had a higher food and water intake.

**Table 1:** Changes in body weight and records of food and water intake of SAMP8 mice supplemented with combined extracts.

**Group** **(*n* = 8)^a^^,^^b^**	**Body weight (gm)**		**Food intake** **(g/day)**	**Water consumption** **(ml/day)**
	**Initial**	**Final**		
Positive control	30.13 ± 0.50	29.17 ± 0.42	5.63 ± 0.08	5.60 ± 0.07
Control	29.67 ± 0.66	27.43 ± 0.43	6.08 ± 0.13	5.69 ± 0.09
Low dose	30.09 ± 0.38	28.28 ± 0.51	5.90 ± 0.10	5.51 ± 0.11
Medium dose	29.08 ± 0.55	27.69 ± 0.49	5.76 ± 0.08	5.56 ± 0.07
High dose	29.01 ± 0.40	27.62 ± 0.34	5.83 ± 0.08	5.54 ± 0.10

a Data are expressed as the mean ± S.E.M. (n = 8) and analyzed by one-way ANOVA.

b Data points were not significantly different (p > 0.05) from each other according to ANOVA. Positive control: CORT (40 mg/kg/day) + Fluoxetine (20 mg/kg/day). Control: CORT (40 mg/kg/day). Low dose: CORT (40 mg/kg/day) + combined extracts of chlorella and lion's mane mushroom (contains 0.1 mL chlorella extract concentrate + 6 mg lion's mane mushroom extract concentrate). Medium dose: CORT (40 mg/kg/day) + combined extracts of chlorella and lion's mane mushroom (contains 0.2 mL chlorella extract concentrate + 12 mg lion's mane mushroom extract concentrate). High dose: CORT (40 mg/kg/day) + combined extracts of chlorella and lion's mane mushroom (contains 0.4 mL chlorella extract concentrate + 24 mg lion's mane mushroom extract concentrate).

### Behavioral tests

#### OFT

The OFT can be used to evaluate the spontaneous activity of animals in a new environment. Animal behavior such as running away or staying in the same place may occur due to fear and stimulation when facing a new environment. The OFT allows for the study of animal performance in terms of locomotion, directed exploratory activity, and emotional state (anxiety) (27, 40). The specific evaluation approach is as follows. When mice with depression symptoms tend to move closer to the peripheral area than normal mice, they are unwilling to remain in the central zone or stay in the corner. When mice traverse the center block with increased frequency and stay there of a long time, they show a reduction in depression and increased exploration ability. The numbers of crossings, stay time, distances traveled, and speeds of movement in the central zone, of SAMP8 mice administered CORT (40 mg/kg/day) and treated with complex extract of chlorella and lion's mane mushroom for 21 consecutive days are shown in Table 2. The results showed that the number of crossings in the positive control group and the treatment groups tended to increase, yet there were no significant differences compared with the control group. Moreover, the stay time and distance traveled (as refers to the total movement distance into the central area) were significantly higher in the positive control group and the medium-, and high-dose treatment groups, compared to the control group (*p* < 0.05). The movement speed was significantly higher only in the positive control group compared with the control group (*p* < 0.05). In a study conducted on mice injected with 20 mg/kg/day of CORT for 5 weeks, a significant reduction in 5 min stays in the central zone in the CORT group compared to the control group was observed (41). Furthermore, during the 3 weeks of continuous injection of 40 mg/kg/day of CORT in mice, a trend toward an increase in the number of crossings of the central zone in 3 min in the CORT group compared to the control group was observed (37).

**Table 2:** Evaluation of the benefits of combined extracts in mice by the open field test (OFT) and the forced swimming test (FST).

	**Group (*n* = 8) ^a^^,^^b^**	**Positive control**	**Control**	**Low dose**	**Medium dose**	**High dose**
Open field test	Frequency	11.38 ± 1.16	9.50 ± 1.34	10.25 ± 0.82	10.75 ± 1.08	11.25 ± 0.56
	Duration time (sec)	17.63 ± 1.13	12.13 ± 1.20	14.75 ± 1.69	16.63 ± 1.85	17.25 ± 1.45
	Distance traveled (mm)	691 ± 110.51	371.71 ± 66.74	447.05 ± 56.74	627.85 ± 72.00	663.38 ± 88.65
	Velocity (mm/s)	29.85 ± 4.15	18.18 ± 1.93	18.84 ± 1.36	19.25 ± 2.12	20.20 ± 3.14
Forced swimming test	Immobility time (sec)	207.58 ± 12.32	254.83 ± 13.81	228.35 ± 12.41	216.45 ± 8.17	210.51 ± 10.89

a Data are expressed as the mean ± S.E.M. (n = 8) and analyzed by one-way ANOVA.

b Data points were not significantly different (p > 0.05) from each other according to ANOVA. Positive control: CORT (40 mg/kg/day) + Fluoxetine (20 mg/kg/day). Control: CORT (40 mg/kg/day). Low dose: CORT (40 mg/kg/day) + combined extracts of chlorella and lion's mane mushroom (contains 0.1 mL chlorella extract concentrate + 6 mg lion's mane mushroom extract concentrate). Medium dose: CORT (40 mg/kg/day) + combined extracts of chlorella and lion's mane mushroom (contains 0.2 mL chlorella extract concentrate + 12 mg lion's mane mushroom extract concentrate). High dose: CORT (40 mg/kg/day) + combined extracts of chlorella and lion's mane mushroom (contains 0.4 mL chlorella extract concentrate + 24 mg lion's mane mushroom extract concentrate).

In brief, according the above, continuous administration of CORT reduces the stay time and the frequency of crossings in the central region, similar to the results obtained in the current study. Hence, the positive control and the medium-, and high-dose treatment groups showed evidence of increased time spent in the central sector, which implies effective improvement in depression, greater mobility in the central zone, and improved exploratory activity in mice.

#### FST

The FST provides a method for evaluating the degree of depression. Depression severity was determined by observing the escape behavior of mice under adverse conditions. Normal mice would struggle in the water, but when their efforts were unsuccessful, they would behave desperately, floating in the water and giving up swimming. The experimental procedure involved video recording to observe the mice's behavior during the experiment and to measure the duration of mobility and immobility. The longer the duration of immobility, the more desperate the mice were. The FST results showed (Table 2) that the mice displayed a significant reduction in time spent immobile during the procedure in the positive control group and medium- and high-dose treatment groups, when compared to the control group (*p* < 0.05). According to previous studies, mice were significantly more immobile in the FST after 3 weeks of continuous CORT treatment (5, 42). It was reported that CORT treatment of mice significantly prolonged immobility in the FST while attenuating behavioral responses in the OFT. In contrast, the administration of catalpol (20 mg/kg for 21 days) significantly reversed CORT-induced depressive behavior in Kunming mice (25). The results of the present study showed the same phenomenon, whereby the administration of antidepressant drugs, as well as medium- and high-doses of combined extracts of chlorella and lion's mane mushroom, was able to reduce both the time that mice gave up struggling and floated on the water surface, which indicated that they were able to alleviate the behavioral manifestations of despair.

### Biochemical analysis of blood

The results of blood biochemical analysis are shown in Table 3. There were significant decreases in triglycerides and BUN in the positive control group and the group treated with a combination of chlorella and lion's mane mushroom extracts, in comparison with the control group (*p* < 0.05). In addition, the analysis showed a decreasing trend in glucose, total cholesterol, GOT, GPT, and creatinine in the positive control group and the complex-treated groups, compared with the control group. However, there were no significant differences in total protein and albumin among all groups. It has been reported that mice injected with 40 mg/kg/day of CORT for 3 consecutive weeks showed increased blood TG, TC, HDL-C, and LDL-C concentrations and a trend of increasing blood glucose concentrations (37).

**Table 3:** Investigation of biochemical analysis of blood profiles in mice treated with combined extracts.

**Group (*n* = 8)^a^^,^^b^**	**Positive control**	**Control**	**Low dose**	**Medium dose**	**High dose**
Albumin (g/dL)	3.04 ± 0.06	3.10 ± 0.08	3.01 ± 0.08	3.12 ± 0.09	3.06 ± 0.08
Glucose (mg/dL)	114.25 ± 4.20	127.38 ± 5.96	121.00 ± 7.65	116.00 ± 7.27	118.63 ± 6.91
Total Cholesterol (mg/dL)	130.88 ± 4.31	141.38 ± 4.93	136.50 ± 3.34	132.75 ± 4.66	131.38 ± 6.32
Triglyceride (mg/dL)	140.25 ± 3.12	167.50 ± 3.15	148.38 ± 4.70	144.13 ± 5.16	142.50 ± 4.81
Total Protein (g/dL)	5.26 ± 0.08	5.30 ± 0.05	5.39 ± 0.08	5.34 ± 0.06	5.33 ± 0.09
GOT (U/L)	82.25 ± 4.95	96.50 ± 2.43	89.38 ± 6.32	85.50 ± 7.12	82.88 ± 4.50
GPT (U/L)	50.63 ± 5.51	61.75 ± 3.53	53.25 ± 3.71	52.38 ± 6.22	56.88 ± 5.79
BUN (mg/dL)	32.63 ± 1.41	40.63 ± 0.94	36.88 ± 1.48	34.75 ± 1.82	35.63 ± 1.67
Creatinine (mg/dL)	0.27 ± 0.02	0.32 ± 0.01	0.30 ± 0.02	0.29 ± 0.02	0.31 ± 0.01

a Data are expressed as the mean ± S.E.M. (n = 8) and analyzed by one-way ANOVA.

b Data points were not significantly different (p > 0.05) from each other according to ANOVA. Positive control: CORT (40 mg/kg/day) + Fluoxetine (20 mg/kg/day). Control: CORT (40 mg/kg/day). Low dose: CORT (40 mg/kg/day) + combined extracts of chlorella and lion's mane mushroom (contains 0.1 mL chlorella extract concentrate + 6 mg lion's mane mushroom extract concentrate). Medium dose: CORT (40 mg/kg/day) + combined extracts of chlorella and lion's mane mushroom (contains 0.2 mL chlorella extract concentrate + 12 mg lion's mane mushroom extract concentrate). High dose: CORT (40 mg/kg/day) + combined extracts of chlorella and lion's mane mushroom (contains 0.4 mL chlorella extract concentrate + 24 mg lion's mane mushroom extract concentrate).

According to previous studies, SD rats with high cholesterol dietary-induced hypercholesterolemia were treated with 100 mg/kg BW lion's mane mushroom extract for 4 weeks showed a significant reduction in blood total cholesterol concentration (43). According to Chovancikova and Simek (44), administration of a high-fat diet with 1% chlorella to CD1 mice for 10 weeks showed a significant reduction in TG and TC in the blood and liver. In brief, it was observed that CORT administration in mice resulted in increased lipid concentrations and elevated blood glucose levels, which were similar to the results in the literature; simultaneous administration of antidepressants and the combined extracts of chlorella and lion's mane mushroom caused a decrease in lipid and blood glucose levels. It was indicated that the combination of chlorella and lion's mane mushroom combined extracts could improve the catabolic effects of CORT.

### Factors correlated with the central nervous system in the brain

#### Serotonin

Serotonin, otherwise known as 5-Hydroxytryptamin (5-HT), is one of the monoamine neurotransmitters in the central nervous system. Acting principally as a neurotransmitter in the synaptic gap, 5-HT is related to mood regulation, behavioral inhibition and mood stabilization, and is a transmitter of mental feelings such as happiness, peace of mind, and satisfaction. The serotonin hypothesis suggests that disorders of the 5-HT system and its components play an active role in the origin of depression (45). It has been suggested that high levels of 5-HT production regulate serotonin neuron clusters by reducing the expression of serotonin transporter and monoamine oxidase A in serotonergic cells via vitamin D (46). An imbalance in the serotonin system in the brain often leads to insomnia, which in turn leads to psychiatric disorders (such as depression and agitation) and, in severe cases, Alzheimer's disease (47). The results of a between-group comparison of brain serotonin (Table 4) revealed an upward trend in the serotonin concentration in the control group; meanwhile, the serotonin concentration in the positive control group and the treatment groups showed a decreasing trend, but it did not reach statistical difference. The results showed that 21 days of CORT administration (40 mg/kg/day) resulted in a significant increase in the 5-HT concentration in the hippocampal cortex of mice (48). Additional evidence suggests that CORT given continuously to rats for 21 days decreased the sensitivity of the prefrontal cortical serotonin receptor (5-HT1A), along with increased prefrontal cortical serotonin concentrations (49). Therefore, it was hypothesized that the increase in serotonin synthesis in the brains of animals with CORT-induced depressive behavior might be caused either by the individual's resistance to excessive stress or by the feedback regulation produced by corticosterone. The results obtained in this study were similar to the above publications concerning the use of CORT to induce depression in SAMP8 mice.

**Table 4:** Effects of combined extracts on the levels of factors correlated to the central nervous system in brain of mice.

**Group (*n* = 8) ^a^^,^^b^**	**Positive control**	**Control**	**Low dose**	**Medium dose**	**High dose**
Serotonin (%)	92.50 ± 4.88	100.00 ± 8.59	96.24 ± 6.97	94.02 ± 6.70	95.64 ± 10.08
Dopamine (%)	94.45 ± 10.90	100.00 ± 6.76	98.55 ± 5.75	98.53 ± 10.34	99.42 ± 6.86
BDNF (%)	141.97 ± 8.73	100.00 ± 9.40	114.09 ± 12.68	118.84 ± 5.51	122.81 ± 8.56

a Data are expressed as the mean ± S.E.M. (n = 8) and analyzed by one-way ANOVA.

b Data points were not significantly different (p > 0.05) from each other according to ANOVA. Positive control: CORT (40 mg/kg/day) + Fluoxetine (20 mg/kg/day). Control: CORT (40 mg/kg/day). Low dose: CORT (40 mg/kg/day) + combined extracts of chlorella and lion's mane mushroom (contains 0.1 mL chlorella extract concentrate + 6 mg lion's mane mushroom extract concentrate). Medium dose: CORT (40 mg/kg/day) + combined extracts of chlorella and lion's mane mushroom (contains 0.2 mL chlorella extract concentrate + 12 mg lion's mane mushroom extract concentrate). High dose: CORT (40 mg/kg/day) + combined extracts of chlorella and lion's mane mushroom (contains 0.4 mL chlorella extract concentrate + 24 mg lion's mane mushroom extract concentrate).

#### Dopamine

Dopamine is one of the neurotransmitters in the central nervous system, and its main effects are related to pleasure and desire, which can govern human actions, motivation, emotions, memory function, and adaptive behavior. It can make people feel excited, give them a desire to emerge, fill them with energy and curiosity, make them emotionally happy, and facilitate a sense of accomplishment. When the concentration of inter-synaptic neurotransmitters is unbalanced, behaviors such as depression, suppressed desire, etc. occur with ease (50). A between-group comparison of brain dopamine concentrations is shown in Table 4. In the control group, the dopamine concentration in the brain was higher than that in the other groups, whereas in the positive control group, the dopamine concentration in the brain decreased following the administration of antidepressant drugs to the mice. In addition, the dopamine concentration in mice of the treatment group was lower than that of mice the control group, but the difference did not reach statistical significance.

According to a study, 21 days of corticosterone administration (20 mg/kg) in mice decreased the expression of glucocorticoid receptors in the cerebral cortex, resulting in dysfunction of the glucocorticoid receptor system, and increased dopamine release from the prefrontal cortex induced by high K^+^ (51). Moreover, another study found that depressive animal behavior was correlated with an increased prefrontal cortical dopamine concentration (52, 53). In this study, it was observed that the dopamine concentration in the brain was similar to those reported in the publications literature.

#### BDNF

BDNF has several functions in the central nervous system, including regulation of nerve cell development, survival, synthesis of synapses, and the transmission of neurotransmitters, which are intracellular messages in the brain. In recent years, various studies have found that BDNF concentrations in the brain and other tissues of depressed patients and animal models were significantly lower than those in normal ones; thus, BDNF has been suggested to be related to the pathological mechanism of depression, where it could be an important therapeutic target for depression (25).

In mice of the positive control group, which were given CORT followed by antidepressant therapy, a significant increase in the BDNF concentration in the brain was observed (*p* < 0.05) (Table 4). Fascinatingly, a similar significant improvement to that in the positive control group was observed in the groups treated with chlorella and lion's mane mushroom combined extract (*p* < 0.05). Many previous studies found that exogenous corticosterone administration caused a decrease in the levels of BDNF mRNA in the hippocampus (54, 55). The continuous administration of CORT (40 mg/kg) for 21 days to rats significantly reduced the protein expression of BDNF in the hippocampal cortex of their brains, while the concurrent administration of an antidepressant (Fluoxetine, 10 mg/kg) improved the concentration of BDNF in the hippocampal cortex (56). In this study, the results were similar to those of the abovementioned studies, which showed that the BDNF concentration in the brains of mice decreased with CORT treatment, although the situation improved with treatment via Fluoxetine and combined extracts (from chlorella and lion's mane mushroom).

The goal of preventive medicine is to promote health and achieve the effect of aging and disease prevention by improving the intake of antioxidant-rich dietary substances (12). Thus, in animal models, high CORT levels contribute to the overproduction of ROS, resulting in increased lipid peroxidation and antioxidant enzyme activity in the brain (1, 25). According to the above results, it was observed that supplementation with combined extracts (from chlorella and lion's mane mushroom) improved the capability of the antioxidant defense system in the body and inhibited the production of peroxides, presumably due to the presence of carotenoids and phenolic compounds in the extracts. Overproduction of amyloid beta-protein (Aβ) causes learning memory deficits (47), and administration of the abovementioned combined extracts might have improved the amyloid deposition in the brain of SAMP8 mice. In general, to our knowledge, there is no information on the effect of chlorella on the deposition of amyloid proteins, but effectively raising antioxidant defenses and reducing the damage caused to nerve cells by oxidative stress might reduce the deposition of amyloid proteins and improve memory loss.

## Conclusions

In summary, this study aimed to observe the effect of different doses of chlorella and lion's mane mushroom combined extracts on depression behavior. All groups of mice were administered CORT (40 mg/kg/day) for 21 days to induce depression *in vivo*. The OFT results showed that the positive control group, and medium- and high-dose extract groups were significantly improved in terms of prolonging the stay in the central zone (*p* < 0.05) and increasing the distance traveled (*p* < 0.05). In terms of FST, the above three groups were able to significantly reduce the immobility time as compared to the control group (*p* < 0.05). The results of blood biochemical analysis showed that the TG and BUN levels in the blood raised by CORT were reduced significantly (*p* < 0.05) in the positive control group, and medium-, and high-dose treatment groups. All three of the above treatments increase BDNF levels. To our knowledge, this is the first validation of the antidepressant-like effects of combined extracts of chlorella and lion's mane mushroom in a CORT injection-induced mouse model of depression, which may lead to the development of a new antidepressant dietary supplement. Hence, this study provides a theoretical framework for future research and application of the combined extracts of chlorella and lion's mane mushroom to prevent and treat depression.

## Data availability statement

The raw data supporting the conclusions of this article will be made available by the authors, without undue reservation.

## Ethics statement

The animal study was reviewed and approved by Committee on Animal Research, Providence University, under code 20170512-A01.

## Author contributions

M-YC, J-HH, and M-JH: conceptualization. Y-JC, M-DY, L-HL, and W-CL: data curation. C-HC, C-HY, T-YT, and W-CL: investigation. J-KT and RJ-cH: methodology. P-HH and P-HL: writing—original draft. P-HL and M-FW: writing—review and editing.

## Funding

This research was financially supported by China Grain Products Research and Development Institute, Taiwan.

## Conflict of interest

The authors declare that the research was conducted in the absence of any commercial or financial relationships that could be construed as a potential conflict of interest.

## Publisher's note

All claims expressed in this article are solely those of the authors and do not necessarily represent those of their affiliated organizations, or those of the publisher, the editors and the reviewers. Any product that may be evaluated in this article, or claim that may be made by its manufacturer, is not guaranteed or endorsed by the publisher.
